# Estimating the Roll Angle for a Two-Wheeled Single-Track Vehicle Using a Kalman Filter

**DOI:** 10.3390/s22228991

**Published:** 2022-11-20

**Authors:** Tzu-Yi Chuang, Xiao-Dong Zhang, Chih-Keng Chen

**Affiliations:** 1 Department of Vehicle Engineering, National Taipei University of Technology, Taipei 10608, Taiwan; 2 School of Information and Mechatronics Engineering, Ningde Normal University, Ningde 352100, China

**Keywords:** two-wheeled vehicle, IMU, Roll angle estimation, Kalman filter

## Abstract

This study determines the roll angle for a two-wheeled single-track vehicle during cornering. The kinematics are analyzed by coordinate transformation to determine the relationship between the measured acceleration and the acceleration in the global coordinate. For a measurement error or noise, the state space expression is derived. Using the theory for a Kalman filter, an estimator with two-step measurement updates estimates the yaw rate and roll angle using the acceleration and angular velocity signals from an IMU sensor. A bicycle with relevant electronic products is used as the experimental object for a steady turn, a double lane change and a sine wave turn in real time to determine the effectiveness of the estimator. The results show that the proposed estimator features perfect reliability and accuracy and properly estimates the roll angle for a two-wheeled vehicle using IMU and velocity.

## 1. Introduction

Two-wheeled single-track vehicles include motorcycles and bicycles and are widely used in cities due to their convenience and high mobility. Four-wheeled vehicles are stable when stationary, but two-wheeled vehicles are not. Therefore, it is necessary to design an active safety system for such vehicles.

When a two-wheeled vehicle turns, it achieves a great roll angle to ensure the balance of forces. According to previous studies on motorcycle dynamics [1], roll angle is a crucial variable, as it determines vehicle behavior, which plays an important role in the research and development of active safety systems. However, it is difficult to accurately determine the roll angle directly using traditional sensors. Therefore, estimating the roll angle when a two-wheeled single-track vehicle is turning has great research value [2,3].

Previous studies have used video and state estimation methods to determine roll angles. A.P. Teerhuis et al. [4] presented a simplified analytic dynamic model of a motorcycle, comparing it to an extended multi-body model. Those authors also used an Extended Kalman Filter (EKF) to obtain signals related to the lateral dynamics of the motorcycle. Nehaoua et al. [5] used a sliding mode observer with an unknown parameter input for a vehicular dynamic model to determine the roll angle. However, this method required accurate vehicle parameters and skill in terms of evaluating the rider’s posture and ability to control the machine. Lot Roberto et al. [6] used a simplified vehicle dynamics model and an Extended Kalman Filter (EKF). Kinematic parameters, such as speed and the angular velocity in the x- and z-directions, were measured. The simplified mathematical model made it possible to make real-time estimations, and the experimental results were accurate.

Corbetta et al. [7] used the three-axis angular velocity from an Inertial Measurement Unit (IMU) and a vehicular kinematics model to determine the roll angle using an Unscented Kalman Filter (UKF). The estimation results were similar to those for real vehicle test experiments, but the UKF was shown to be more robust to inaccurate initial conditions. Savaresi et al. [8] used the frequency separation method for estimations and used the same sensor signal as that described in the study by Lot et al. [6]. Using the integral value of the angular velocity in the x-direction for high-frequency estimations, the low-frequency signal was calculated using a vehicle kinematics model. The two signals were combined to obtain the final estimated value.

Boniolo et al. [9,10] used two sets of low-cost gyroscopes to measure the *x*-, *y*-, and *z*-axis angular velocities. The roll angle was calculated using the ratio of the *y*- or *z*-axis angular velocity to the *x*-axis angular velocity. By combining the angular velocities of the *x*-axis and the *y*- or *z*-axes by frequency separation method, the authors of [8] could estimate the roll angle. This method avoids errors if the roll angle is too great; however, the configuration is very complicated.

Similarly to the study by Savaresi et al. [8], Schwab et al. [11] used the integral value of the roll angular velocity and the *y*- and *z*-axis angular velocities from the IMU to determine the roll angle using the frequency separation method. This method was susceptible to the effect of IMU noise when driving in a straight line. Ahmed et al. [12] used the third axis acceleration and the three-axis angular velocity signal, as measured by an IMU and a gyroscope, to calculate the lateral and vertical acceleration. They then determined the vehicle’s attitude (pitch and roll angles) using a Kalman filter. Sanjurjo et al. [13] demonstrated a roll angle estimator that used an EKF and the angular rate. However, the multibody dynamics of the study motorcycle were simplified, so the estimation was not accurate. P-M Damon et al. [14] used an unknown input observer to estimate lateral motorcycle dynamic states and to reconstruct unknown inputs in real riding scenarios. In addition, Ding Yao et al. [15,16] applied an intelligence algorithm and a graph neural network to design an adaptive filter. The application of self-supervised methods provided a good reference for roll angle estimates.

The roll angle can also be estimated using image recognition [17]. Schlipsing et al. [18] compared four estimation methods, including image recognition and an IMU complementary filter. The results showed that a Kalman filter that used IMU signals was very accurate, and that estimates that used image recognition were affected by the driving environment, such as sky and weather conditions. These studies used different methods to determine the roll angle, but the applied sensors were expensive and too numerous, and accuracy varied under different driving conditions. Motivated by the above discussion, this paper proposes an estimation with two-step measurements based on a kinematic model and Kalman filter theory, which is an improvement of work reported in [11,12,13]. The major contribution of our research is the improvement of estimation accuracy.

This paper is organized as follows. Section 2 determines the kinematic characteristics using coordinate transformation. Section 3 derives the state space expression that includes the measurement values and the error. Section 4 describes the proposed estimator, which uses two-step measurement updates to determine the yaw rate and roll angle. Section 5 describes an experiment that used a bicycle with relevant electronic products to determine the performance of the proposed estimator. Section 6 presents the conclusions.

## 2. Kinematic Analysis

**Figure 1:**
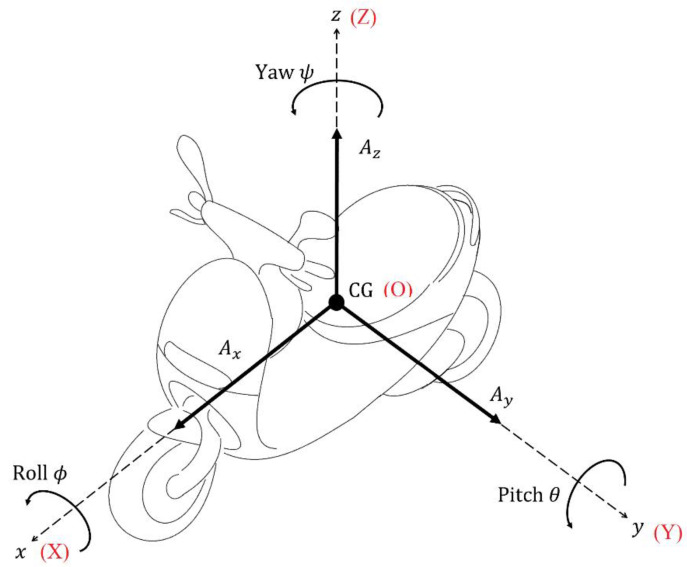
Vehicle coordinate and parameters.

This study uses a coordinate transformation method to describe the kinematic parameters for a vehicle. The moving coordinate CG-xyz is fixed to the vehicle body, as shown in Figure 1. The origin is located at the center of gravity, the *x*-axis is in the direction of forward motion, the *z*-axis is vertical to the vehicle’s motion and the *y*-axis is determined using the right-hand rule. The global coordinate O-XYZ is fixed on the ground and does not change when the vehicle moves. The moving and the global coordinates initially move in the same direction, so the attitude is determined by transforming the global coordinate into a moving one.

In the global coordinate, accelerations along the three axes comprise longitudinal acceleration $A_{x}$, lateral acceleration $A_{y}$ and vertical acceleration $A_{z}$, as shown in Figure 1. For the moving coordinate system, there are three accelerations along the axes of the moving coordinates $a_{xm}$, $a_{ym}$, $a_{zm}$, which are measured by an IMU sensor on the vehicle. The IMU sensor measures angular velocity signals $\omega_{xm}$, $\omega_{ym}$, $\omega_{zm}$. All signals from the IMU apply to the moving coordinate system.

**Figure 2:**
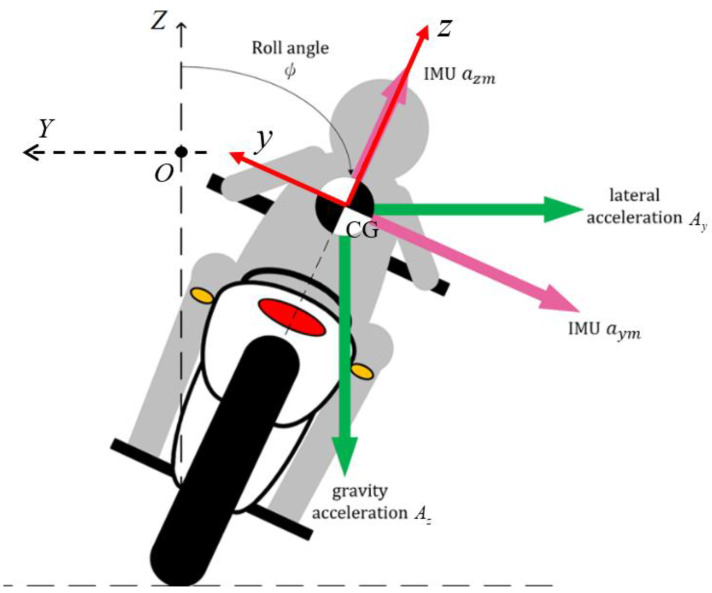
Dynamics of a turning motorcycle.

When a motorcycle turns, it achieves roll angle ϕ, as shown in Figure 2, to compensate for the centrifugal force and to maintain balance.

Using coordinate transformation, the kinematic parameter that transforms the relationship between the global and the moving coordinate is determined using Equation (1). This is also the geometric relationship between the measured acceleration signals and the acceleration in the global coordinates.

(1) $$\left[\begin{array}{c} A_{x}\\ A_{y}\\ A_{z} \end{array}\right]=\left[\begin{array}{ccc} 1 & 0 & 0\\ 0 & \cos\phi & -\sin\phi \\ 0 & \sin\phi & \cos\phi \end{array}\right]\left[\begin{array}{c} a_{xm}\\ a_{ym}\\ a_{zm} \end{array}\right]$$

Equation (1) is used to derive Equations (2)–(4) as:

(2) $$A_{x}=a_{xm}$$

(3) $$A_{y}=a_{ym}\cos\phi -a_{zm}\sin\phi$$

(4) $$A_{z}=a_{ym}\sin\phi +a_{zm}\cos\phi$$

When a motorcycle turns stably, the lateral acceleration is expressed as:

(5) $$A_{y}=v_{x}\dot{\psi }\;$$

where $v_{x}$ is longitudinal velocity and $\dot{\psi }$ is the yaw rate.

## 3. State Space Expression

To determine the roll angle, the measured roll rate $\omega_{xm}$ is integrated to obtain the preliminary value for roll angle $\phi_{g}$. This integration occurs in Equation (6):

(6) $$\phi_{g,k}=\phi_{g,k-1}+\omega_{xm,k}\Delta t\;$$

where Δt is the sample time and $\phi_{g,k-1}$ is the preliminary value at the last time step. However, the angular velocity signal from the IMU is sensitive to measurement noise, so the integrated value may not be accurate.

By transposing Equations (3) and (4), the roll angle $\phi_{a}$ that is calculated using acceleration signals is expressed as s:

(7) $$\phi_{a}=\sin^{-1}\frac{a_{ym}\cos\phi_{a}-A_{y}}{a_{zm}}$$

(8) $$\phi_{a}=\cos^{-1}\frac{a_{zm}\sin\phi_{a}+A_{y}}{a_{ym}}\;$$

Equation (8) can diverge if the vehicle is moving straight, because the roll angle and the lateral acceleration are approximately zero. Therefore, Equation (7) is used for the measurement update phase for the Kalman filter.

If the vehicle is cornering steadily, the lateral acceleration is equal to the product of the longitudinal velocity and the yaw rate. It is also presumed that the roll angle does not change significantly in a short time. As such, $\phi_{a,k}$ at the right side is replaced with the value at last time step, $\phi_{a,k-1}$, and Equation (7) is rewritten to express roll angle $\phi_{a,k}$ as:

(9) $$\phi_{a,k}=\sin^{-1}\frac{a_{ym}\cos\phi_{a,k-1}-v_{x}\dot{\psi }}{a_{zm}}$$

In Equation (9), the longitudinal velocity, the acceleration and yaw rate signals, the estimated roll angle and the value at last time step gives the final value of the roll angle, $\phi_{a,k}$. However, when cornering, the *z*-axis angular velocity $\dot{\psi }$ is proportionally different to the $\omega_{zm}$ component of the IMU signal because there is a roll angle. The measured signal may also contain noise that affects the accuracy of the estimation, so the yaw rate is calculated initially and then the roll angle is calculated on that basis.

If the sum of the real acceleration value is equal to that for the measured value from the IMU sensor, then squaring and adding Equations (3) and (4) gives Equation (10):

(10) $$A_{y}{}^{2}+A_{z}{}^{2}={\left(a_{ym}\cos\phi -a_{zm}\sin\phi \right)}^{2}+{\left(a_{ym}\sin\phi +a_{zm}\cos\phi \right)}^{2}$$

Simplifying and organizing Equation (10) gives:

(11) $$A_{y}{}^{2}+A_{z}{}^{2}=a_{ym}{}^{2}+a_{zm}{}^{2\;}$$

Substituting Equation (5) into Equation (11) gives Equation (12):

(12) $${\left(v_{x}\dot{\psi }\right)}^{2}+A_{z}{}^{2}=a_{ym}{}^{2}+a_{zm}{}^{2\;}$$

Transposing the terms and solving the square root of Equation (12) allows the absolute value for the yaw rate to be expressed as:

(13) $$\left|{\dot{\psi }}_{a}\right|=\sqrt{\frac{a_{ym}{}^{2}+a_{zm}{}^{2}-A_{z}{}^{2}}{v_{x}{}^{2}}}$$

The sign of the yaw rate depends on the turning direction. Therefore, the sigmoidal membership Function is added to Equation (13). This function slightly reduces the noise in the signals by adjusting the curve for the function. If the vehicle is travelling on a flat road, the vertical acceleration $A_{z}$ is equal to the force of gravity, g, so the yaw rate is expressed as:

(14) $${\dot{\psi }}_{a}=\mathrm{sigmf}\left(\omega_{zm}\right)\sqrt{\frac{a_{ym}{}^{2}+a_{zm}{}^{2}-g^{2}}{v_{x}{}^{2}}}$$

The roll angle is calculated by integrating the *x*-axis angular velocity signal from the IMU assuming a zero pitch angle; however, the signals from the IMU are easily affected by noise, so an error in value *d* describes the difference between the integrated roll angle and the true value, as shown in Equation (15):

(15) $$d=\phi_{g}-\phi$$

where the d is roll angle error.

Error value *e* is also added to describe the difference between the *z*-axis angular velocity signal from the IMU and the true yaw rate, as shown in Equation (16):

(16) $$e=\omega_{zm}-\dot{\psi }$$

where the *e* is error in the yaw rate.

Using Equations (15) and (16), the system state is defined as $x_{k}={\left[\begin{array}{ccc} \begin{array}{cc} \phi_{g} & d \end{array} & \dot{\psi } & e \end{array}\right]}^{T}$. The system input is defined as $u_{k}={\left[\begin{array}{cc} \omega_{xm,k} & \omega_{zm,k} \end{array}\right]}^{T}$. The output is defined as:

(17) $$y_{1,k}={\dot{\psi }}_{a,k}$$

(18) $$y_{2,k}=\phi_{a,k}$$

As there is disturbance in the real system, the system noise and measured noise must be added to the state space expression. The system state equation and the measured equation are then:

(19) $$x_{k}=A_{r}x_{k-1}+B_{r}u_{k}+W_{k-1}$$

(20) $$y_{1,k}=H_{1}x_{k}+V_{1,k}$$

(21) $$y_{2,k}=H_{2}x_{k}+V_{2,k}$$

Equation (19) is the state equation and Equations (20) and (21) are measurement equations. $A_{r}$ denotes the system matrix and $B_{r}$ is the control matrix, which are shown in Equation (22). $W_{k-1}$ is the system noise vector and $H_{1}$ and $H_{2}$ are measurement matrices, shown in Equation (23); $V_{1,k}$ and $V_{2,k}$ are measurement noise.

(22) $$A_{r}=\left[\begin{array}{cccc} 1 & 0 & 0 & 0\\ 0 & 1 & 0 & 0\\ 0 & 0 & 0 & -1\\ 0 & 0 & 0 & 1 \end{array}\right]\;,\;B_{r}=\left[\begin{array}{cc} \Delta t & 0\\ 0 & 0\\ 0 & 1\\ 0 & 0 \end{array}\right]$$

(23) $$H_{1}=\left[\begin{array}{cccc} 0 & 0 & 1 & 0 \end{array}\right],\;\;\;H_{2}=\left[\begin{array}{cccc} 1 & -1 & 0 & 0 \end{array}\right]\;$$

## 4. Estimator Design

The roll angle significantly affects the stability of two-wheeled vehicles, but it is difficult to accurately determine directly using a sensor because of system disturbances or measurement noise. Our estimator for the roll angle uses a Kalman filter. The yaw rate is determined initially, and this value is used to determine the roll angle.

According to state space expression, the Kalman estimator state is defined as: ${\hat{x}}_{r}={\left[\begin{array}{ccc} \begin{array}{cc} {\hat{\phi }}_{g} & \hat{d} \end{array} & \hat{\dot{\psi }} & \hat{e} \end{array}\right]}^{T}$. The estimator model is expressed as:

(24)  x ^r,k=Arx^r,k−1+Brur,k 

(25) $${\hat{\dot{\psi }}}_{k}=H_{1}{\hat{x}}_{r,k}\;$$

(26) $${\hat{\phi }}_{k}=H_{2}{\hat{x}}_{r,k}$$

The filter recurrence formula for yaw rate $\hat{\dot{\psi }}$ is:

(27) $${\hat{x}}_{r,k}^{+}={\hat{x}}_{r,k}^{-}+K_{1,k}(y_{1,k}-H_{1}{\hat{x}}_{r,k}^{-})$$

where ${\hat{x}}_{r,k}^{-}$ is a one-step estimated state that is calculated using Equation (24), i.e., the gain matrix, which is calculated as:

(28) $$K_{1,k}=P_{k}^{-}H_{1}^{T}{\left(H_{1}P_{k}^{-}H_{1}^{T}+R_{1}\right)}^{-1}$$

where $P_{k}^{-}$ is the prediction error covariance matrix that is calculated as:

(29) $$P_{k}^{-}=A_{r}P_{k-1}^{++}A_{r}^{T}+Q_{r}$$

where $R_{1}$ and $Q_{r}$ are the respective covariance matrices for measurement noise $V_{1,k}$ and system noise $W_{k}$, which are written as:

(30) $$R_{1}=\mathrm{Var}\;(\;V_{1,k}\;),\;\;\;Q_{r}=\mathrm{Var}\;(\;W_{k}\;)$$

Filter error variance matrix $P_{k}^{+}$ is calculated using Equation (31). This is then used as the prediction error covariance matrix to determine the roll angle.

(31) $$P_{k}^{+}=(I-K_{1,k}H_{1})P_{k}^{-}$$

When state estimation vector ${\hat{x}}_{r,k}^{+}$ has been determined, the estimated yaw rate value $\hat{\dot{\psi }}$ may be determined using Equation (25). The value for $\hat{\dot{\psi }}$ is used to calculate $y_{2,k}$ to determine the roll angle.

The filter recurrence formula for the estimated roll angle $\hat{\phi }$ is:

(32) $${\hat{x}}_{r,k}^{++}={\hat{x}}_{k}^{+}+K_{2,k}(y_{2,k}-H_{2}{\hat{x}}_{k}^{+})$$

One-step estimated state ${\hat{x}}_{k}^{+}$ is the last variable required to estimate the yaw rate. $K_{2,k}$ is the gain matrix that is calculated as:

(33) $$K_{2,k}=P_{k}^{+}H_{2}^{T}{\left(H_{2}P_{k}^{+}H_{2}^{T}+R_{2}\right)}^{-1}$$

where $R_{2}$ is variance matrix for measurement noise $V_{2,k}$, which is expressed as:

(34) $$R_{2}=\mathrm{Var}\;(\;V_{2,k}\;)$$

The filter error variance matrix $P_{k}^{++}$ is used to calculate the prediction error covariance matrix $P_{k}^{-}$ for the next cycle using Equation (29):

(35) $$P_{k}^{++}=(I-K_{2,k}H_{2})P_{k}^{+}$$

Using the state estimation vector ${\hat{x}}_{r,k}^{++}$ calculated using Equation (32), the estimated roll angle $\hat{\phi }$ is calculated using Equation (26).

**Figure 3:**
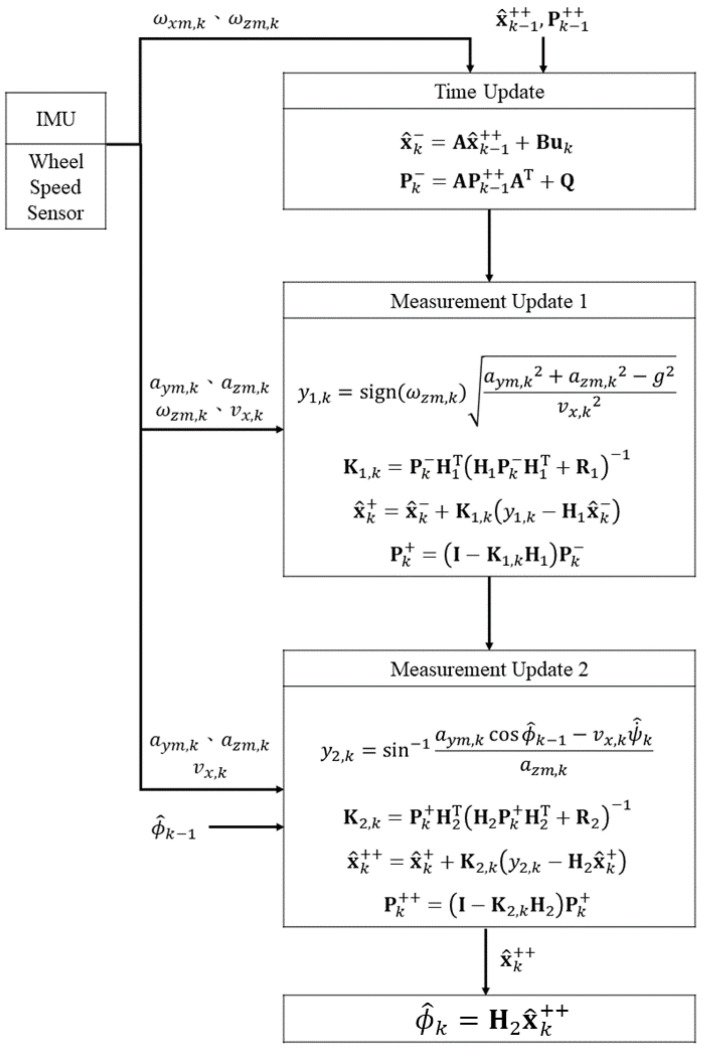
Flow chart for estimating the roll angle.

A flow chart for estimating roll angle n is shown in Figure 3. There are three phases: a time update and the first and second measurement updates.

In the time update phase, an integration model is used to calculate the roll angle. The signals that are used are the angular velocities along the *x*- and *z*-axes that are measured by the IMU. The one-step estimated state ${\hat{x}}_{k}^{-}$ and the prediction error covariance matrix $P_{k}^{-}$ are expressed using an upper index [−].

The first measurement update estimates the yaw rate. For a zero-pitch angle and longitudinal acceleration, the quadratic sum of the lateral and vertical acceleration is the same as that of the *y* and *z*-axis acceleration signals from the IMU. Estimated state ${\hat{x}}_{k}^{+}$ and covariance matrix $P_{k}^{+}$ are expressed using the upper index [+] after the first measurement update.

The second measurement update estimates the roll angle. The estimated yaw rate value, the lateral acceleration, the vertical acceleration, the longitudinal velocity and the estimated roll angle from the last step are used to update the state. The result is ${\hat{x}}_{k}^{++}$; this is used to calculate the estimated roll angle using Equation (26).

## 5. Experiment and Results

**Figure 4:**
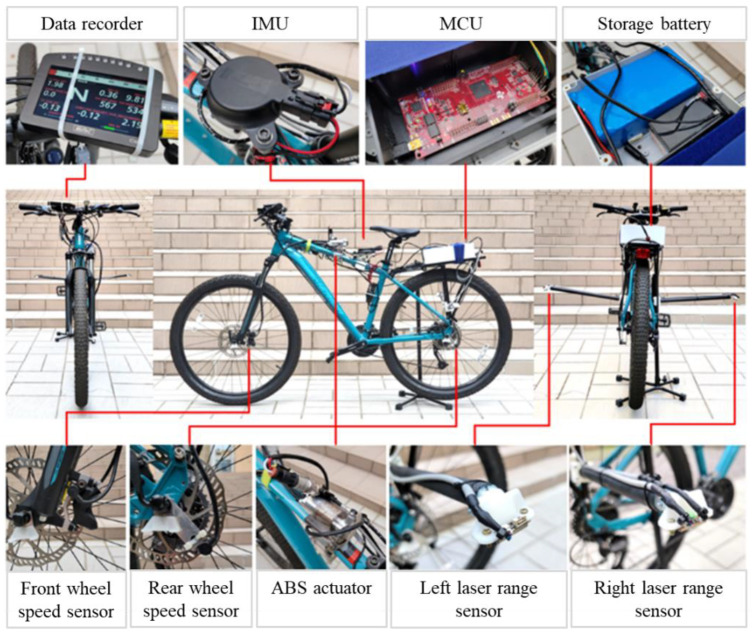
Hardware installed on the experimental vehicle.

This study used practical tests to validate the feasibility and robustness of the estimator that is described in Section 4. Although motorcycles and bicycles are different, both are two-wheeled single-track vehicles. Therefore, the kinematic model of a bicycle, without considering the influence of mass and inertia, is similar to that of motorcycle. The proposed estimation was designed based on a kinematic model. Therefore, a bicycle experiment was set up to verify the effectiveness of the estimation model, and electronic hardware was installed on the bicycle to construct an experimental platform. The bicycle with the hardware is shown in Figure 4. A g data recorder, an IMU, a Microprogrammed Control Unit (MCU), a 12 V battery, wheel speed sensors, laser range sensors using the time of flight (ToF) method and an ABS actuator were fitted to the bicycle. A data recorder was used to record the test data for analysis. An ABS actuator measured the longitudinal velocity.

**Figure 5:**
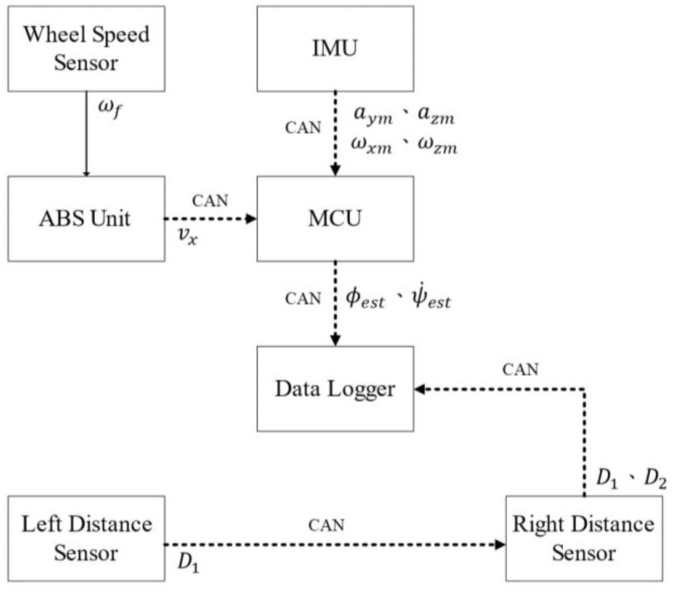
Experimental scheme.

The experimental setup is shown in Figure 5. The longitudinal velocity, measured by the ABS unit, and the measured acceleration and angular velocity signals from the IMU were transmitted to the MCU using a controller area network (CAN). The estimated roll angle was transmitted to the data recorder by a CAN bus.

**Figure 6:**
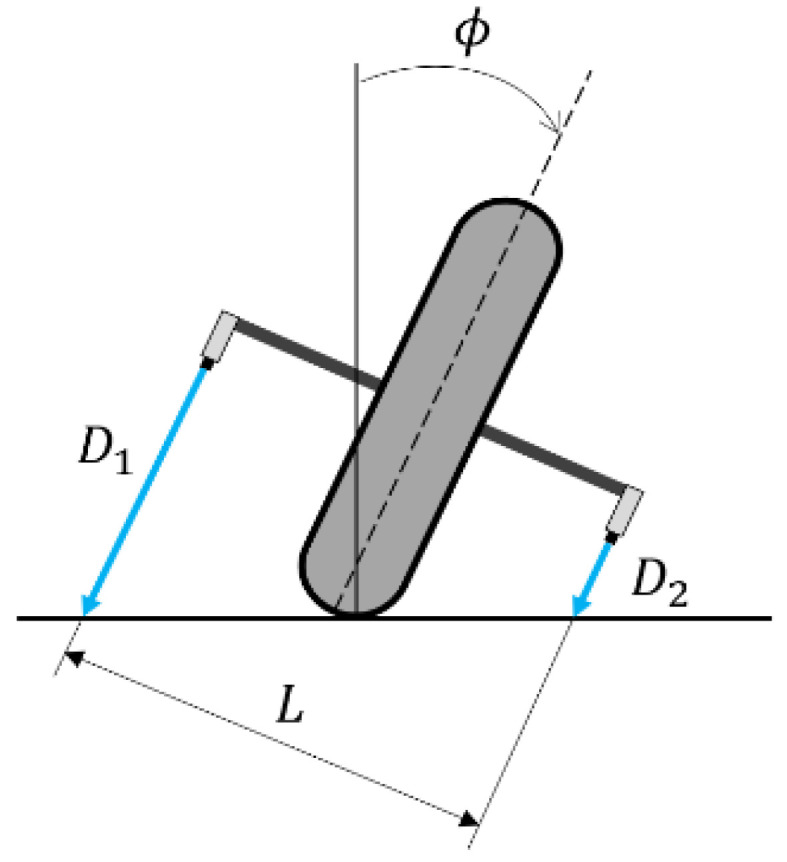
Range sensors on both sides of the rear wheel. *D*_1_—Distance signal from left laser sensor; *D*_2_—Distance signal from right laser sensor, *L*—distance between two laser sensors, *φ*—the true value for the roll angle.

The true value for the roll angle was calculated using the distance signals from the laser sensors that were mounted on both sides of the rear wheel, as shown in Figure 6. Equation (36) was used to calculate the roll angle based on data from the laser sensors.

(36) $$\phi =\tan^{-1}\left(\frac{D_{1}-D_{2}}{L}\right)$$

The covariance matrix for system noise $Q_{r}$ and the covariance matrix for measurement noise in the first and the second measurements update are represented as $R_{1}\;\mathrm{and}\;R_{2}$; these parameters were defined by trial and error using Equation (37):

(37) $$Q_{r}=\left[\begin{array}{cccc} 1 & 0 & 0 & 0\\ 0 & 0.0001 & 0 & 0\\ 0 & 0 & 10 & 0\\ 0 & 0 & 0 & 0.1 \end{array}\right],\;R_{1}=\left[1000\right],\;R_{2}=\left[100\right]$$

Tests validated the performance of the proposed estimator for three driving modes: steady cornering, a double lane change and sine wave turning. During testing, the data recorder recorded the true values for the roll angle, the estimated value from the Kalman filter without yaw rate correction and the estimated value from the proposed estimator. Errors in the estimations were then determined.

### 5.1. Steady Turning

**Figure 7:**
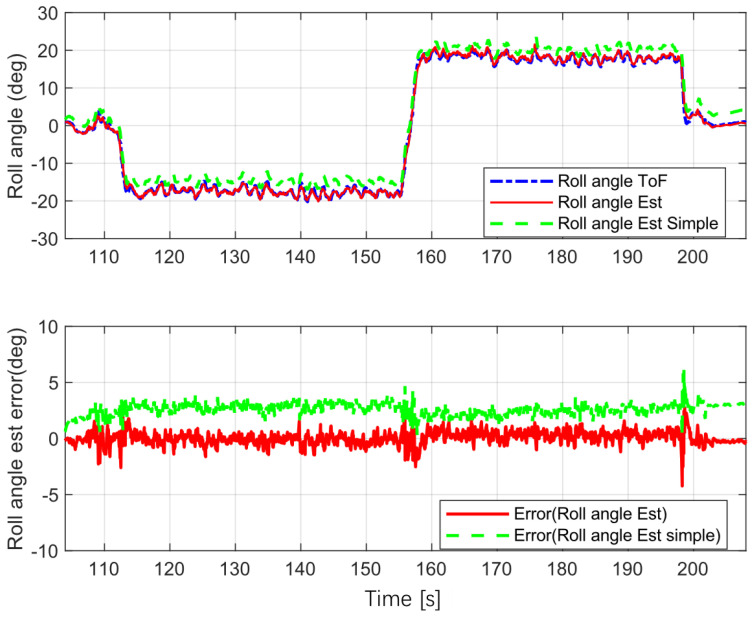
Steady cornering.

This test used a fixed velocity and a fixed center for turning in a circle. The radius was 5 m. Five revolutions to the left and to the right were used; the test results are shown in Figure 7.

Roll angle ToF is the ground true value, roll angle EST Simple is the estimated value from the Kalman filter without yaw rate correction and roll angle EST is the value estimated by the proposed estimator.

### 5.2. Double Lane Change (DLC)

**Figure 8:**
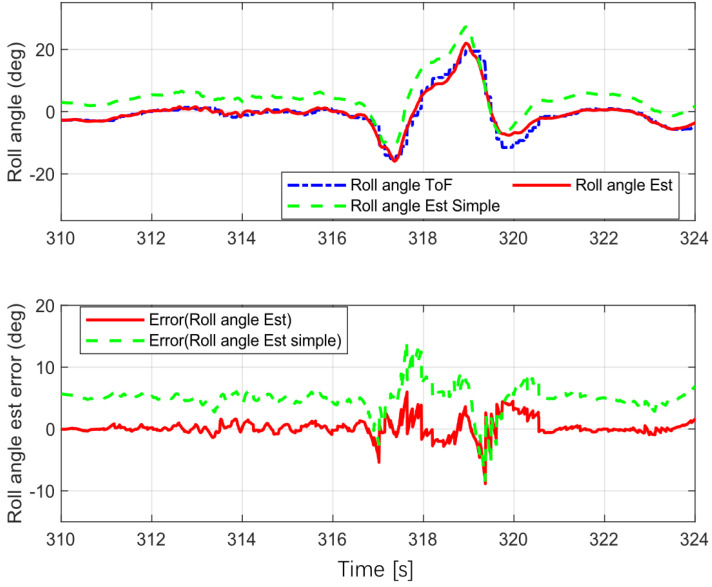
Double lane change.

This procedure was used to simulate avoiding an unexpected obstacle on the road. The entry velocity of the bicycle was 20 km/h. The test results are shown in Figure 8.

### 5.3. Sine Wave Turning

**Figure 9:**
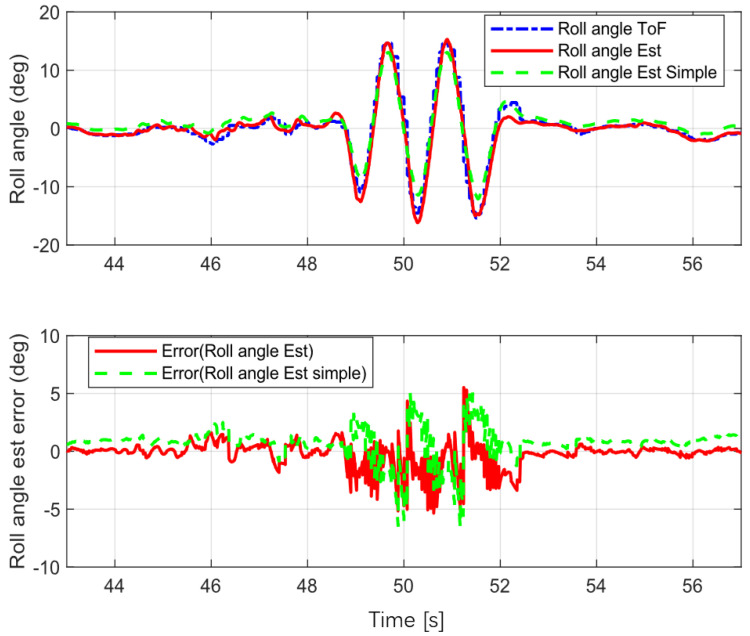
Sine wave turning.

The S-turn mode tested the stability of the proposed estimator during high-frequency turns. The test vehicle changed direction 6 times in about 5 s over a lateral distance of 2.5 m. The velocity for this procedure was about 10 to 20 km/h. The test results are shown in Figure 9.

The error statistics for the estimation are shown in Table 1. The mean absolute error (MEA) for the proposed estimation was less than that for the simple version (without yaw rate correction), and the same was true of the root mean square error (RMSE) for the proposed estimator. These results show that the proposed estimator was more accurate than the simple version for various scenarios, and that it estimated the vehicle roll angle accurately.

## 6. Conclusions

This study proposes a roll angle estimator for a two-wheeled single-track vehicle. Vehicle kinematics were used to determine the relationship between the measured value and the vehicle motion, and the measurement noise and system noise were used to derive the state space expression. Using the theory of a Kalman filter, a roll angle estimator with two-step measurement update t estimated the yaw rate and the roll angle. This architecture reduced the yaw rate error from the IMU and provided more accurate estimations of the roll angle.

An experimental platform was used for tests for three typical modes of movement. A comparison of the results of the proposed system and a simple version estimator showed that the former estimated the roll angle more accurately in each scenario, confirming its feasibility and robustness.

## Figures and Tables

**Table 1:** Error statistics for roll angle estimation.

		MAE	RMSE
Steady Turning (Figure 7)	Simple	2.6043	2.6537
	Proposed	0.4311	0.5692
DLC (Figure 8)	Simple	5.1574	5.4322
	Proposed	0.8461	1.3768
Sine Wave (Figure 9)	Simple	1.2070	1.5060
	Proposed	0.7020	1.1613

## Data Availability

Not applicable.
